# Older People Hospitalized for COVID-19: Prognostic Role of Multidimensional Prognostic Index and Other Prognostic Scores

**DOI:** 10.3390/jcm12020594

**Published:** 2023-01-11

**Authors:** Luca Carruba, Maria Armata, Giusy Vassallo, Carlo Saccaro, Carla Di Palermo, Chiara Giannettino, Laura Cilona, Rossella Capitummino, Nicola Veronese, Ligia J. Dominguez, Mario Barbagallo

**Affiliations:** 1 Department of Health Promotion, Mother and Child Care, Internal Medicine and Medical Specialties “G. D’Alessandro”, University of Palermo, 90127 Palermo, Italy; 2 Faculty of Medicine and Surgery, University of Enna “Kore”, 94100 Enna, Italy

**Keywords:** COVID-19, multidimensional prognostic index, comprehensive geriatric assessment, mortality, prognosis

## Abstract

During the SARS-CoV-2 pandemic, frailty and patients’ poor outcomes seem to be closely related. However, there is no clear indication of the significance of this connection and the most adequate risk index in clinical practice. In this study, we compared a short version of MPI (multidimensional prognostic index) and other two prognostic scores for COVID-19 as potential predictors of poor patient outcomes. The patients were consecutively enrolled in the hospital of Palermo for COVID-19. The accuracy of Brief-MPI, 4C score and COVID-GRAM score in points was evaluated using the area under the curve (AUC) with 95% CI, taking mortality or sub-ICU admission as outcome. The study included 112 participants (mean age 77.6, 55.4% males). During a mean of 16 days of hospitalization, Brief-MPI significantly increased by 0.03 ± 0.14 (*p* = 0.04), whilst COVID-GRAM did not. Brief-MPI, 4C score and COVID-GRAM scores had good accuracy in predicting negative outcomes (AUC > 0.70 for all three scores). Brief-MPI was significantly associated with an increased mortality/ICU admission risk, indicating the importance of multidimensional impairment in clinical decision-making with an accuracy similar to other prognostic scores commonly used in COVID-19 study, providing information regarding domains for which interventions can be proposed.

## 1. Introduction

The outbreak of coronavirus disease of 2019 (COVID-19), caused by Severe Acute Respiratory Syndrome coronavirus 2 (SARS-CoV-2), has resulted in a global pandemic [1,2]. Since the start of the COVID-19 pandemic, older patients have been identified as one of the most vulnerable categories and a high-risk group with increased risk of complications and death from COVID-19 [3]. This pandemic has resulted in significant challenges worldwide, and our understanding of this disease continues to evolve, despite the efficacy of vaccination.

The epidemiological data available indicated that COVID-19 could be considered as an emerging geriatric condition [4], as its prevalence and mortality are higher in older patients compared to young adults [5]. Prognosis of older COVID-19 patients, particularly when affected by frailty, could be bad as frailty is a prominent risk factor for severe disease and death from COVID-19 [6]. In order to avoid progression towards an irreversible disability, it could be beneficial to recognize and manage the most frail subjects early. This could also result in a more extensive evaluation of older patients in terms of risk of adverse outcomes due to impaired ability to cope with daily or acute stressors, as evaluated by the comprehensive geriatric assessment (CGA) [7]. For this reason, in geriatric patients, besides a stratification based on age and comorbidities determinants, it could also be necessary to identify a multidimensional prognostic score that involves a functional, social and psychosocial evaluation, and is easy to apply and able to predict a patient’s outcome, such as intensive or sub-intensive care unit (ICU) admission and mortality.

The Multidimensional Prognostic Index (MPI) is a widely used prognostic index for estimating both short- and long-term mortality, easily derived on information gathered from a CGA [8,9,10,11,12,13]. Initially developed and validated in hospitalized older patients [8], a series of multicenter studies, involving more than 60,000 older participants across different settings and medical conditions, reported that the MPI is an accurate and well calibrated tool for predicting mortality and other negative health outcomes [8,9,10,11,12,14]. MPI shows a high validity, reliability and feasibility for the management of older persons with different degrees of complexity [15]. The MPI is currently one of the most commonly used tools to identify and measure frailty both in the hospital and other healthcare settings [16,17]. MPI was also used during the COVID-19 pandemic for better refining the prognosis of older people in nursing homes [18] or hospitals [19,20]. However, studies comparing the prognostic accuracy of MPI versus other tools commonly used in clinical practice, such as the 4C score or COVID-GRAM score, were not available.

Given this background, the aim of this study is to compare a brief version of the MPI, 4C and COVID-GRAM scores in order to find a tool that can be easily applied in clinical practice as a potential predictor of negative outcomes such as mortality and admission to sub-ICU, with characteristics typical of a multidimensional evaluation.

## 2. Materials and Methods

### 2.1. Data Source and Patients

We included patients hospitalized in Internal Medicine or Geriatrics Wards in the University Hospital (Policlinico) ‘P. Giaccone’ in Palermo, Sicily, Italy with an age ≥ 65 years and a diagnosis of SARS-CoV-2 infection confirmed by positive nasal-pharyngeal swab. The patients were verbally informed regarding the aims of the study; informed consent was collected verbally for hygienic reasons. The only exclusion criterion was the inability to understand the aims of the study.

All older patients were directly admitted from the local Emergency Department to our wards. The patients were enrolled whether vaccinated or not. The percentage of the patients vaccinated was 70.5%, most with three doses (37.5% of the entire sample).

The study was approved by the Local Ethical Committee during the session of the 28th April 2021 (number 04/2021), in the context of the COMEPA (COVID-19 Medicina Policlinico Palermo) study [21].

### 2.2. Data Collection

All participants included in the study completed the COVID-GRAM score and the Brief-MPI at admission and at discharge/transfer to other wards. The admission parameters were gathered through patient/family interviews and clinical documentation: age, gender, clinical history, medication history and current pathologies such as congestive heart failure, hypertension, respiratory disease, renal impairment, endocrine/metabolic disease, vascular disease, malignant disease, anemia and dementia. Laboratory tests were required on the first day of hospitalization and were repeated before discharge/transfer, according to clinical presentation.

### 2.3. COVID-GRAM Score

The COVID-GRAM score was initially developed in China and aims to predict the onset of a critical COVID-19 illness, defined as admission to the intensive care unit, invasive mechanical ventilation or death [22].

The score applied ten variables, commonly measured on admission to the hospital:

- Age;
- Unconsciousness;
- Hemoptysis;
- Dyspnea;
- Number of comorbidities (includes diabetes, hypertension, heart disease, cerebral vascular disease, kidney disease, cancer and immunodeficiency);
- Cancer history;
- Chest radiography abnormality;
- Neutrophil-to-lymphocyte ratio;
- Direct bilirubin;
- Lactate dehydrogenase.

These variables were used to stratify patients with a low, medium or high risk of developing a critical illness. Low risk of critical illness ≤1.7%, medium risk = 1.7% to <40.4% and high risk ≥40.4% [22].

### 2.4. The 4C Score

The 4C Mortality Score was derived from a prospective observational cohort study based upon positively tested COVID-19 patients admitted to 260 hospitals in England, Scotland and Wales. The score includes eight variables consisting of age, sex, number of comorbidities, respiratory rate, peripheral oxygen saturation, Glasgow coma scale score, urea level and C-reactive protein. The score, ranging from 0 to21, predicts in-hospital mortality from low risk to very high risk [23].

### 2.5. Brief-MPI

The Brief-MPI is a prognostic tool that had a good agreement with the standard version of the MPI [24]. The Brief-MPI includes eight domains, as does the full version, consequently keeping its multidimensional value:

1. Activities of daily life, derived from the activities of daily living (ADL) test [25], from which three were selected, i.e., dressing, feeding and self-control over urination and defecation;
2. Instrumental activities of daily living, derived from the instrumental ADL (IADL) [26], from which three were selected, i.e., using the telephone, taking medications and shopping;
3. Cognitive assessment, using the Short Portable Mental Status Questionnaire (SPMSQ) [27], with questions regarding computation ability and evaluation of personal and temporal orientation;
4. Mobility assessment, evaluated using the Barthel mobility index [28] and including the ability to move from bed to chair, walk and climb stairs;
5. Nutritional assessment, evaluated with the Mini-Nutritional Assessment Short Form [29] with the following three questions: body mass index (<21 or ≥30 kg/m^2^), loss of appetite in the last three months and weight loss in the last three months;
6. Comorbidities as evaluated using the Cumulative Illness Rating Scale (CIRS) [30] that uses a 5-point ordinal scale (score 1–5) to estimate the severity of pathology in each of 13 systems, including cardiac, vascular, respiratory, eye-ear-nose-throat, upper and lower gastrointestinal, hepatic, renal, genitourinary, musculoskeletal, skin disorder, nervous system, endocrine–metabolic and psychiatric behavioral disorders. Based on the ratings, the Comorbidity Index (CIRS-CI) score, which reflects the number of concomitant diseases, was derived from the total number of categories in which moderate or severe levels (grade from 3 to 5) of disease were identified (range from 0 to 13). In the brief version of the MPI, we used the CIRS to determine the pathologies for which at least one medication was needed;
7. Number of drugs in use;
8. Cohabitation status.

The first seven domains had a dichotomic response (yes/no or right/wrong). The value of cohabitation status was 0 for individuals who lived with the family, 0.5 for those institutionalized, and 1 for those who lived alone. Each domain received a risk rating (low risk = 0, moderate risk 0.5, high risk = 1). Compared to the standard version, the brief version requires less than 5 min, making] this tool ideal for COVID-19 wards. The Brief-MPI is freely available at https://multiplat-age.it/index.php/en, accessed on 20 November 2022.

### 2.6. Outcomes

The outcomes of interest of our analysis were mortality, recorded using medical records and death certificates, and admission to a sub-ICU ward. This latter structure was managed by specialists in respiratory medicine and admitted patients with elevated needs in terms of oxygenation without orotracheal intubation. None of our patients were admitted to an ICU.

### 2.7. Statistical Analysis

Data are reported as means with standard deviations (SDs) for continuous variables and as percentages for categorical parameters, by patients alive vs. patients dead/admitted to sub-ICU wards. The parameters were compared, at the baseline evaluation, using independent *t* tests or chi-square tests (considering the Fisher’s exact test as appropriate). Brief-MPI, 4C score and COVID-GRAM scores (in points and in percentages) were compared using matched *t* tests, and changes were calculated as the difference between discharge/death and hospital admission (delta). Cases with missing data regarding Brief-MPI and COVID-GRAM scores were not considered.

The strength of the association between the Brief-MPI, divided in two classes, and death/sub-intensive care admission was analyzed using a Cox’s regression analysis, adjusted for sex, reporting the results as hazard ratios (HRs) with 95% confidence intervals (CIs). The accuracy of Brief-MPI, 4C and COVID-GRAM scores in points was evaluated using the area under the curve (AUC) with 95% CI, taking mortality or sub-ICU admission as outcome.

Significance was accepted if *p* < 0.05, and all tests were two-tailed. All analyses were performed using SPSS 25.0 for Windows (SPPS Inc., Chicago, IL, USA).

## 3. Results

Among the 246 older people initially screened, the study included a total of 112 patients affected by COVID-19. The mean age was 77.6 ± 10.3 and 44.6% were women. Among the 134 patients excluded, 34 had insufficient data regarding the Brief-MPI and the other prognostic scores of interest. During the 90-day follow-up period, of the 112 patients enrolled in the study, 19 died or were admitted to sub-ICU care. The mean age of dead/sub-intensive care group was 80.3 ± 10.3 years and 57.9% were women; these parameters did not differ from their counterparts (Table 1). On the contrary, patients in the dead/sub-ICU care group were characterized by significantly higher values in Brief-MPI (*p* = 0.001), 4C (*p* = 0.002) and COVID-GRAM (*p* < 0.0001) scores at the time of admission, as shown in Table 1. In particular, patients who died or were admitted to sub-intensive care were more frequently non-autonomous in IADL (*p* = 0.001) and non-self-sufficient in ADL (*p* < 0.001). Furthermore, patients in this group had higher scores on the Barthel mobility index (*p* = 0.003) and higher risk of pressure sores (ESS, *p* < 0.0001) compared to their counterparts.

Table 2 reports the changes in the Brief-MPI parameters and in COVID-GRAM score between hospital discharge and admission. During an average period of 16 days of hospitalization, the Brief-MPI values significantly increased by 0.03 ± 0.14 (*p* = 0.04) points; on the contrary, the COVID-GRAM did not undergo any changes. Among the domains of the Brief-MPI included, only the nutritional and disability domains showed a significant worsening between the two evaluations.

**Figure 1:**
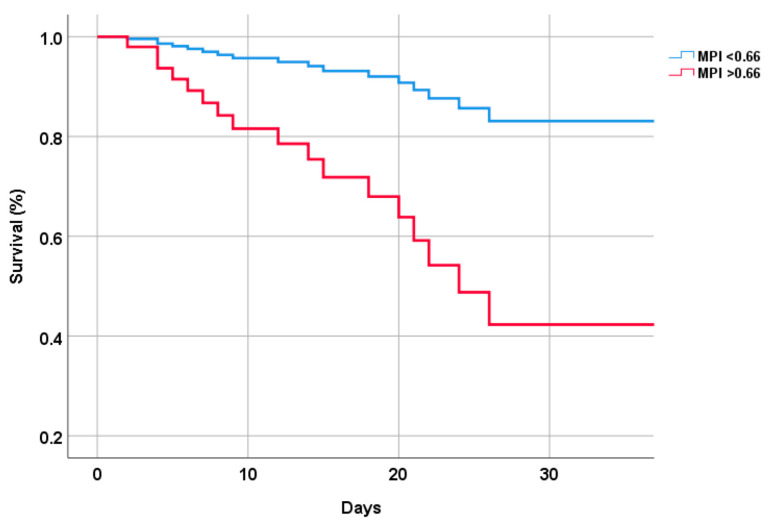
Survival curve by multidimensional prognostic index values.

Figure 1 shows the survival curves according to the Brief-MPI values at the baseline evaluation. In the analyses adjusted for sex, compared to patients with a Brief-MPI ≤ 0.66, a Brief-MPI value > 0.66 upon admission to the ward was associated with a significant risk of death or admission to sub-intensive care (HR = 8.52; 95% CI: 4.24–16.78; *p* < 0.0001). In particular, the mean survival rate 16 days after admission was approximately 75%.

**Figure 2:**
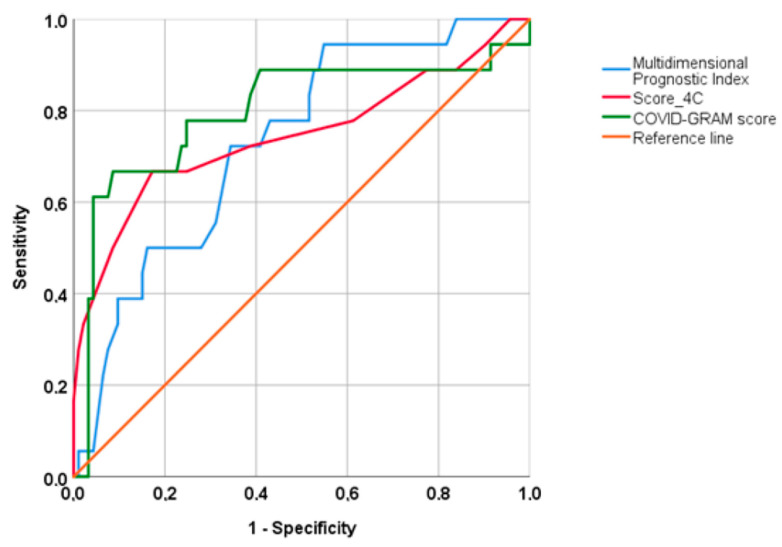
Accuracy of multidimensional prognostic index, 4C score and COVID-GRAM.

However, as shown in Figure 2, both the Brief-MPI (AUC 0.754; 95% CI: 0.629–0.880; *p* = 0.001), the 4C score (AUC 0.746; 95%CI: 0.594–0.897; *p* = 0.001) and the COVID-GRAM score (AUC 0.826; 95% CI: 0.700–0.951; *p* < 0.0001) were good predictors of unfavorable outcomes in older patients, without any significant differences (*p* = 0.44 vs. COVID-GRAM and *p* = 0.54 vs. 4C score).

## 4. Discussion

In this observational study involving 112 patients with an age ≥65 years, with a diagnosis of a SARS-CoV-2 infection, we found that Brief-MPI was significantly associated with an increased mortality/sub-ICU admission risk, indicating the importance of multidimensional impairment in clinical decision-making in older people.

The topic of prognosis in COVID-19 research has been extensively studied. This pandemic, in fact, showed the need for prognostic tools able to identify older people at higher risk of mortality and unfavorable outcomes [31]. Unfortunately, most prediction model studies were poorly reported and of low quality, as they often reported limited predictive performances [32]. At the same time, there is still a paucity of prognostic tools based on CGA that evaluate the impact of COVID-19 in older subjects, even if some recent reports suggested that the impact of the COVID-19 pandemic on frailty condition is largely independent of the direct effect of the virus [33]. Consistently, growing evidence brings attention to the burden of the indirect effects of COVID-19 (i.e., psychological distress, cognitive impairment, malnutrition and physical inactivity), which are reflected in multidimensional well-being [34]. Therefore, it is reasonable to think, particularly during this pandemic, that only a CGA-based approach is qualified to really capture and track the changes in frailty condition.

This study adds to the literature the comparison of three scores for the prognosis of hospitalized patients affected by COVID-19, i.e., one derived from a multidimensional evaluation of the older subjects and the other one commonly used in COVID-19, i.e., the COVID-GRAM Score. Our results indicate that the multidimensional impairment is more important than the evaluation based only on COVID-19 parameters in older hospitalized people, even if the two tools are similar in terms of accuracy.

Therefore, our current work can highlight the importance of having an adequate risk index for quickly identifying frail individuals and helping the physician in daily clinical practice.

Another important finding is that, during the hospitalization, we observed a significant worsening in MPI score between discharge and admission. Even if the change had a mean of only 0.03, it could be of clinical relevance as in other studies a similar change was associated with a higher risk of unfavorable outcomes [9,35]. The main driver for these findings is the worsening in the disability item that was observed in other studies conducted on hospitalized older people [36] and, often due to the physical inactivity observed in this population, this was probably more pronounced in COVID-19 patients [37]. Another domain that presented a significant decline was the nutritional domain, which further remarks the importance of malnutrition in COVID-19 patients. A systematic review, for example, showed that the overall prevalence of malnutrition was 42.1% in hospitalized patients [38]; however, our study indicates that nutritional aspects probably worsened during hospital stay. Finally, we observed that during hospitalization the mean number of medications increased by one drug, indicating that polypharmacy could be another problem upon hospital discharge. It was reported that polypharmacy could be associated with a higher risk of negative outcomes in COVID-19 patients [39].

The findings of our study must be interpreted within the study’s limitations. First, the sample size was relatively small. Second, the incidence of mortality and ICU admission was relatively limited, not permitting the division of these two outcomes. Third, as expected in longitudinal studies involving older people, a consistent portion of the patients included in our analyses had no information regarding the prognostic scores investigated. Excluding participants due to missing data can, unfortunately, introduce an important selection bias in our findings.

## 5. Conclusions

Brief-MPI was significantly associated with an increased mortality/ICU admission risk, indicating the importance of multidimensional impairment in clinical decision-making with an accuracy similar to other prognostic scores commonly used in COVID-19, providing information regarding domains for which interventions can be proposed. We believe that our findings further underline the importance of prognostic factors derived from CGA to better predict negative outcomes in patients affected by COVID-19 in terms of mortality and/or ICU admission.

## Figures and Tables

**Table 1:** Descriptive characteristics at the baseline by survival status.

Factor	Dead/ Sub-Intensive Care (*n* = 19)	Alive (*n* = 93)	*p*-Value
Age (mean, SD)	80.3 (10.3)	77.3 (9.6)	0.22
Female sex (*n*, %)	57.9	41.9	0.20
ADL (mean, SD)	1.0 (1.2)	2.2 (1.1)	<0.0001
IADL (mean, SD)	0.89 (1.24)	1.89 (1.18)	0.001
SPMSQ (mean, SD)	1.32 (1.11)	0.84 (1.04)	0.07
ESS (mean, SD)	11.95 (4.22)	15.57 (3.28)	<0.0001
CIRS (mean, SD)	4.53 (2.32)	3.76 (2.06)	0.15
MNA (mean, SD)	1.26 (1.10)	0.80 (0.98)	0.07
Barthel mobility (mean, SD)	0.58 (1.07)	1.51 (1.36)	0.003
Living alone (*n*, %)	15.8	10.8	0.79
Brief MPI (mean, SD)	0.60 (0.23)	0.42 (0.21)	0.001
COVID-GRAM (%) (mean, SD)	73 (28)	36 (26)	<0.0001
COVID-GRAM (points) (mean, SD)	166 (54)	134 (60)	0.04
4C score (mean, SD)	12.8 (3.4)	10.0 (2.4)	0.002

Abbreviations: SD: Standard Deviation; ADL: Activities Daily Living; IADL: Instrumental Activities Daily Living; SPMSQ: Short Portable Mental Status Questionnaire; ESS: Exton-Smith Scale; CIRS: Cumulative Illness Rating Scale; MNA: Mini Nutritional Assessment; MPI: Multidimensional Prognostic Index.

**Table 2:** Changes in multidimensional prognostic index parameters and in COVID-GRAM score between hospital discharge and admission.

Item	Discharge	Admission	Mean Change (SD)	*p*-Value
ADL	2.03 (1.18)	2.20 (1.07)	−0.16 (0.58)	0.008
IADL	1.84 (1.18)	1.91 (1.16)	−0.08 (0.68)	0.29
SPMSQ	0.88 (1.13)	0.82 (1.01)	0.07 (0.85)	0.46
ESS	15.2 (3.7)	15.6 (3.28)	−0.36 (2.24)	0.13
CIRS	3.83 (1.99)	3.76 (2.06)	0.07 (0.79)	0.39
MNA	0.89 (0.97)	0.80 (0.98)	0.09 (0.45)	0.05
Barthel mobility	1.43 (1.34)	1.51 (1.36)	−0.08 (0.65)	0.27
Drugs	8.51 (3.75)	7.20 (3.74)	1.31 (2.80)	<0.0001
Brief MPI	0.45 (0.26)	0.42 (0.21)	0.03 (0.14)	0.04
COVID-GRAM (%)	64.5 (32.0)	36.7 (26.3)	27.8 (32.2)	0.41
COVID-GRAM (points)	136 (104)	134 (60)	2 (65)	0.78

Abbreviations: SD: Standard Deviation; ADL: Activities Daily Living; IADL: Instrumental Activities Daily Living; SPMSQ: Short Portable Mental Status Questionnaire; ESS: Exton-Smith Scale; CIRS: Cumulative Illness Rating Scale; MNA: Mini Nutritional Assessment; MPI: Multidimensional Prognostic Index.

## Data Availability

Data are available upon reasonable request to the corresponding author.

## References

[1] McIntosh K., Hirsch M.S., Bloom A. (2020). Coronavirus disease 2019 (COVID-19). UpToDate Hirsch MS Bloom.

[2] Chavez S., Long B., Koyfman A., Liang S.Y. (2021). Coronavirus Disease (COVID-19): A primer for emergency physicians. Am. J. Emerg. Med..

[3] Veronese N., Barbagallo M. (2021). Specific approaches to patients affected by dementia and covid-19 in nursing homes: The role of the geriatrician. Ageing Res. Rev..

[4] Lloyd-Sherlock P.G., Kalache A., McKee M., Derbyshire J., Geffen L., Casas F.G.-O. (2020). WHO must prioritise the needs of older people in its response to the covid-19 pandemic. BMJ.

[5] Onder G., Rezza G., Brusaferro S. (2020). Case-fatality rate and characteristics of patients dying in relation to COVID-19 in Italy. JAMA.

[6] Chen X., Mao G., Leng S.X. (2014). Frailty syndrome: An overview. Clin. Interv. Aging.

[7] Veronese N., Custodero C., Demurtas J., Smith L., Barbagallo M., Maggi S., Cella A., Vanacore N., Aprile P.L., Ferrucci L. (2022). Comprehensive geriatric assessment in older people: An umbrella review of health outcomes. Age Ageing.

[8] Pilotto A., Ferrucci L., Franceschi M., D’Ambrosio L.P., Scarcelli C., Cascavilla L., Paris F., Placentino G., Seripa D., Dallapiccola B. (2008). Development and validation of a multidimensional prognostic index for one-year mortality from comprehensive geriatric assessment in hospitalized older patients. Rejuvenation Res..

[9] Pilotto A., Veronese N., Daragjati J., Cruz-Jentoft A.J., Polidori M.C., Mattace-Raso F., Paccalin M., Topinkova E., Siri G., Greco A. (2019). Using the multidimensional prognostic index to predict clinical outcomes of hospitalized older persons: A prospective, multicenter, international study. J. Gerontol. Ser. A.

[10] Cella A., Veronese N., Pomata M., Leslie Quispe Guerrero K., Musacchio C., Senesi B., Prete C., Tavella E., Zigoura E., Siri G. (2021). Multidimensional frailty predicts mortality better than physical frailty in community-dwelling older people: A five-year longitudinal cohort study. Int. J. Environ. Res. Public Health.

[11] Pilotto A., Veronese N., Siri G., Bandinelli S., Tanaka T., Cella A., Ferrucci L. (2021). Association between the Multidimensional Prognostic Index and Mortality during 15 Years of Follow-Up in the InCHIANTI Study. J. Gerontol. Ser. A.

[12] Cruz-Jentoft A.J., Daragjati J., Fratiglioni L., Maggi S., Mangoni A.A., Mattace-Raso F., Paccalin M., Polidori M.C., Topinkova E., Ferrucci L. (2020). Using the Multidimensional Prognostic Index (MPI) to improve cost-effectiveness of interventions in multimorbid frail older persons: Results and final recommendations from the MPI_AGE European Project. Aging Clin. Exp. Res..

[13] Schäfer M., Körber M.I., Vimalathasan R., Mauri V., Iliadis C., Metze C., Ten Freyhaus H., Baldus S., Polidori M.C., Pfister R. (2021). Risk stratification of patients undergoing percutaneous repair of mitral and tricuspid valves using a multidimensional geriatric assessment. Circ. Cardiovasc. Qual. Outcomes.

[14] Pilotto A., Custodero C., Maggi S., Polidori M.C., Veronese N., Ferrucci L. (2020). A multidimensional approach to frailty in older people. Ageing Res. Rev..

[15] Warnier R., Van Rossum E., Van Velthuijsen E., Mulder W., Schols J., Kempen G. (2016). Validity, reliability and feasibility of tools to identify frail older patients in inpatient hospital care: A systematic review. J. Nutr. Health Aging.

[16] Dent E., Martin F.C., Bergman H., Woo J., Romero-Ortuno R., Walston J.D. (2019). Management of frailty: Opportunities, challenges, and future directions. Lancet.

[17] Veronese N., Custodero C., Cella A., Demurtas J., Zora S., Maggi S., Barbagallo M., Sabba C., Ferrucci L., Pilotto A. (2021). Prevalence of multidimensional frailty and pre-frailty in older people in different settings: A systematic review and meta-analysis. Ageing Res. Rev..

[18] Veronese N., Koyanagi A., Stangherlin V., Mantoan P., Chiavalin M., Tudor F., Pozzobon G., Tessarin M., Pilotto A. (2021). Mortality attributable to COVID-19 in nursing home residents: A retrospective study. Aging Clin. Exp. Res..

[19] Custodero C., Cella A., Veronese N., Azzini M., Fimognari F., Mattace-Raso F., Polidori M.C., Sabbà C., Pilotto A. (2020). The Multidimensional Prognostic Index (MPI) for the prognostic stratification of hospitalized older patients with COVID-19: A prospective multicenter observational cohort study. Objectives, study design and expected outcomes (MPI_COVID-19). Geriatr. Care.

[20] Pilotto A., Azzini M., Cella A., Cenderello G., Castagna A., Pilotto A., Custureri R., Dini S., Farinella S.T., Ruotolo G. (2021). The multidimensional prognostic index (MPI) for the prognostic stratification of older inpatients with COVID-19: A multicenter prospective observational cohort study. Arch. Gerontol. Geriatr..

[21] The COMEPA group (2021). COMEPA (COVID-19 Medicina Policlinico Palermo): A study in hospitalized patients. Geriatr. Care.

[22] Liang W., Liang H., Ou L., Chen B., Chen A., Li C., Li Y., Guan W., Sang L., Lu J. (2020). Development and validation of a clinical risk score to predict the occurrence of critical illness in hospitalized patients with COVID-19. JAMA Intern. Med..

[23] Knight S.R., Ho A., Pius R., Buchan I., Carson G., Drake T.M., Dunning J., Fairfield C.J., Gamble C., Green C.A. (2020). Risk stratification of patients admitted to hospital with covid-19 using the ISARIC WHO Clinical Characterisation Protocol: Development and validation of the 4C Mortality Score. BMJ.

[24] Cella A., Veronese N., Custodero C., Castagna A., Cammalleri L.A., Capitano W.M., Solimando L., Carruba L., Sabbà C., Ruotolo G. (2022). Validation of Abbreviated Form of the Multidimensional Prognostic Index (MPI): The BRIEF-MPI Project. Clin. Interv. Aging.

[25] Wallace M., Shelkey M. (2007). Katz index of independence in activities of daily living (ADL). Urol. Nurs..

[26] Lawton M.P., Brody E.M. (1969). Assessment of older people: Self-maintaining and instrumental activities of daily living. Gerontologist.

[27] Pfeiffer E. (1975). A short portable mental status questionnaire for the assessment of organic brain deficit in elderly patients. J. Am. Geriatr. Soc..

[28] Mahoney F.I., Barthel D.W. (1965). Barthel index. Md. State Med. J..

[29] Kaiser M.J., Bauer J.M., Ramsch C., Uter W., Guigoz Y., Cederholm T., Thomas D.R., Anthony P., Charlton K.E., Maggio M. (2009). Validation of the Mini Nutritional Assessment Short-Form (MNA®-SF): A practical tool for identification of nutritional status. JNHA-J. Nutr. Health Aging.

[30] Salvi F., Miller M.D., Grilli A., Giorgi R., Towers A.L., Morichi V., Spazzafumo L., Mancinelli L., Espinosa E., Rappelli A. (2008). A manual of guidelines to score the modified cumulative illness rating scale and its validation in acute hospitalized elderly patients. J. Am. Geriatr. Soc..

[31] Izcovich A., Ragusa M.A., Tortosa F., Lavena Marzio M.A., Agnoletti C., Bengolea A., Ceirano A., Espinosa F., Saavedra E., Sanguine V. (2020). Prognostic factors for severity and mortality in patients infected with COVID-19: A systematic review. PLoS ONE.

[32] Wynants L., Van Calster B., Collins G.S., Riley R.D., Heinze G., Schuit E., Bonten M.M., Dahly D.L., Damen J.A., Debray T.P. (2020). Prediction models for diagnosis and prognosis of covid-19: Systematic review and critical appraisal. BMJ.

[33] Pilotto A., Custodero C., Zora S., Poli S., Senesi B., Prete C., Tavella E., Veronese N., Zini E., Torrigiani C. (2022). Frailty trajectories in community-dwelling older adults during COVID-19 pandemic: The PRESTIGE study. Eur. J. Clin. Investig..

[34] Vernuccio L., Sarà D., Inzerillo F., Catanese G., Catania A., Vesco M., Cacioppo F., Dominguez L.J., Veronese N., Barbagallo M. (2022). Effect of COVID-19 quarantine on cognitive, functional and neuropsychiatric symptoms in patients with mild cognitive impairment and dementia. Aging Clin. Exp. Res..

[35] Volpato S., Daragjati J., Simonato M., Fontana A., Ferrucci L., Pilotto A., Group M.-T.S. (2016). Change in the multidimensional prognostic index score during hospitalization in older patients. Rejuvenation Res..

[36] Sleiman I., Rozzini R., Barbisoni P., Morandi A., Ricci A., Giordano A., Trabucchi M. (2009). Functional trajectories during hospitalization: A prognostic sign for elderly patients. J. Gerontol. Ser. A Biomed. Sci. Med. Sci..

[37] Crisafulli A., Pagliaro P. (2021). Physical activity/inactivity and COVID-19. Eur. J. Prev. Cardiol..

[38] Bedock D., Lassen P.B., Mathian A., Moreau P., Couffignal J., Ciangura C., Poitou-Bernert C., Jeannin A.-C., Mosbah H., Fadlallah J. (2020). Prevalence and severity of malnutrition in hospitalized COVID-19 patients. Clin. Nutr. ESPEN.

[39] Iloanusi S., Mgbere O., Essien E.J. (2021). Polypharmacy among COVID-19 patients: A systematic review. J. Am. Pharm. Assoc..

